# Associations Among Cyberbullying Victimization, Inhibitory Control, Neural Activation of Error Processing, and Mental Health Problems in Adolescents: Neuroimaging, Retrospective Longitudinal Cohort Study Using the Adolescent Brain Cognitive Development Data

**DOI:** 10.2196/75126

**Published:** 2026-02-18

**Authors:** Xuanyu Zhang, Chengyan Xie, Yu Chen, Boyu Qiu

**Affiliations:** 1 School of Health Management, Guangzhou Medical University Guangzhou, Guangdong China; 2 The Third School of Clinical Medicine, Guangzhou Medical University Guangzhou China; 3 Department of Psychiatry, Yale University School of Medicine New Haven, CT United States

**Keywords:** cyberbullying victimization, mental health problems, error processing, functional magnetic resonance imaging, stop signal task

## Abstract

**Background:**

Cyberbullying victimization is prevalent and closely linked to mental health problems. However, existing research, often limited by cross-sectional designs and a focus on direct relationships, has yielded inconsistent results. Furthermore, the biological mechanisms underlying the relationship between cyberbullying victimization and psychopathological outcomes remain largely unclear at present.

**Objective:**

This retrospective cohort study aimed to explore the longitudinal associations among cyberbullying victimization, inhibitory control, brain activation during error processing, and mental health problems among adolescents.

**Methods:**

We curated the clinical, behavioral, and neuroimaging data (551/1186, 46.5% girls; 9-10 years at baseline) from the Adolescent Brain Cognitive Development study, a nationally representative cohort established through school-based probability sampling (selected factors included gender, race/ethnicity, socioeconomic status, and urbanicity). Participants were assessed by the cyberbullying question, the functional magnetic resonance imaging stop signal task for inhibitory control and error processing, and the Child Behavioral Checklist for externalizing and internalizing problems at 2-year (T1) and 4-year follow-up (T2). Linear mixed models were used to examine the retrospective longitudinal associations between these clinical, behavioral, and neuroimaging factors.

**Results:**

Linear mixed models showed that victims of cyberbullying at T1 exhibited significantly greater externalizing problems at T2 (β=0.25, 95% CI 0.06-0.45, *P*_FDR_=.02), but not for internalizing problems (β=–0.01, 95% CI –0.20 to 0.19, *P*_FDR_=.99) or deficits in inhibitory control (Correct Stop Rate: β=–0.02, 95% CI –0.26 to 0.21, *P*_FDR_=.85; Stop Signal Reaction Time: β=–0.07, 95% CI –0.27 to 0.13, *P*_FDR_=.85). Furthermore, cyberbullying victimization at T1 contributed to higher activation in the bilateral superior parietal gyri (left: β=0.36, 95% CI 0.10-0.61, *P*_FDR_=.04; right: β=0.34, 95% CI 0.08-0.59, *P*_FDR_=.04), right inferior parietal gyrus (β=0.32, 95% CI 0.07-0.57, *P*_FDR_=.04), and right posterior cingulate cortex (β=0.34, 95% CI 0.09-0.60, *P*_FDR_=.04) during error processing at T2. However, these neural alterations did not significantly mediate between cyberbullying victimization at T1 and externalizing problems at T2.

**Conclusions:**

This longitudinal functional magnetic resonance imaging study investigates neural correlates of cyberbullying victimization in adolescents. By extending prior research that has relied primarily on cross-sectional or behavioral data, this research demonstrates that this form of victimization is associated with altered neural activation during error processing in later development. The pattern of nonsignificant impairment in inhibitory control and mediation to externalizing problems suggests that these neural impacts may be better characterized by a state of heightened sensitivity and compensatory engagement than by direct damage. Overall, this study points to the error-processing network as a potential target for cognitive interventions and establishes a foundation for further exploration of other neural mechanisms between cyberbullying victimization and mental health outcomes.

## Introduction

### Literature Review

Bullying is a prevalent risk factor for mental health disorders among adolescents [1,2]. In an era characterized by web-based relationships and interactions, cyberbullying has become increasingly common [3,4], which is defined as the repeated and deliberate harm by a perpetrator to a victim through mobile phones, computers, and other electronic devices [5]. A growing number of studies have linked cyberbullying victimization to mental health problems in adolescents, including more internalizing problems [6-8] such as depression [9,10] and anxiety [11,12] as well as more externalizing problems such as aggressive [13,14] and rule-breaking behaviors [15,16].

However, there exists a subset of studies that failed to find the significant effects of cyberbullying victimization on adolescents’ mental health problems [17-20]. Such heterogeneity on this topic may be attributed to differences in experimental design (eg, cross-sectional vs longitudinal study) and variable measurement (eg, different questionnaire assessments) across studies, particularly the tendency to focus solely on direct observational relationships without sufficiently investigating the underlying psychological and physiological mechanisms that may explain how and for whom these effects occur [21]. Therefore, there is a current need for more longitudinal studies that use consistent, well-validated measures and incorporate biological perspectives to clarify the pathways linking cyberbullying victimization to mental health.

Grounded in the stress process model for internalizing problems [22] and the general aggression model for externalizing problems [23], the association between cyberbullying victimization and these psychopathological outcomes may be mediated by inhibitory control and its associated neural mechanisms. Inhibitory control, the ability to suppress inappropriate responses to stimuli [24], is negatively affected by various adverse childhood experiences (ACEs) [25], such as threat [26,27], deprivation [28,29], and abuse [30,31]. Inhibitory control deficits disrupt an individual’s emotional and behavioral regulation, further contributing to internalizing and externalizing problems [32-34]. Given that the cyberbullying victimization in adolescents shares key characteristics with ACEs (ie, exposure to adversity, trauma, stress, or threats) and is closely linked to mental health problems, it is pertinent to explore whether diminished inhibitory control mediates the association between cyberbullying victimization and psychopathological outcomes.

According to the error detection theory [35,36], performance on inhibitory control is closely linked to the brain activation during error processing. Specifically, when individuals fail to inhibit a response, the cingulate cortex typically shows increased activation, as it reflects the perception of errors (ie, failed inhibition) [37,38] and supports subsequent cognitive and behavioral adjustments [39,40]. Moreover, previous studies have shown that ACEs impede the error monitoring circuitry, leading to blunted activation in cingulate cortices during failed inhibition [41,42]. Therefore, we focused on the cingulate cortices as regions of interest (ROIs) to investigate whether or how cyberbullying victimization decreases neural responses and contributes to inhibitory control deficits. The cingulate cortex further interacts with frontal [43,44] and parietal [45,46] cortices during error processing to maintain inhibitory control, which are key regions of the cognitive control network closely linked to information integration, attention regulation, and adaptive behaviors [47,48]. Given that ACEs have been suggested to reduce activation and connectivity within this network [49,50], we also included frontoparietal regions as ROIs to examine potential alterations of brain activation in cyberbullying victims.

Notably, several studies have indicated that adolescents with ACEs may exhibit excessive, rather than insufficient, activation in cingulate, frontal, and parietal cortices during response inhibition and error processing [51-53]. Such brain over-activation within the cognitive control network may reflect heightened sensitivity to errors and hypervigilance to adversity following trauma and stress caused by ACEs [54] and predicts the development of mental health problems such as depression and anxiety [55-57]. Since cyberbullying victimization is also traumatic [5] and stressful [58] for adolescents, it is plausible that a mechanism exists whereby cyberbullying victimization directly mediates excessive activation of cingulate, frontal, and parietal cortices, contributing to psychopathological outcomes.

McLoughlin et al [59] provided initial functional magnetic resonance imaging (fMRI) evidence supporting this potential mechanism. Their findings showed that viewing cyberbullying-related stimuli activated responses in many brain regions (eg, frontal, cingulate, and parietal cortices), closely associated with cognitive and emotional processing [60]. Despite the negative impacts of viewing cyberbullying content, adolescents may also be victims of cyberbullying rather than bystanders. Exploring the specific neural harms of cyberbullying victimization can contribute to a comprehensive understanding of the negative impacts of cyberbullying as well as the development of effective postvictimization interventions. However, based on their review [61] and to our knowledge, no longitudinal studies have investigated the neural alterations after adolescents experienced cyberbullying.

The abilities of inhibitory control and error monitoring improve progressively throughout adolescence, which are essential for maturing adaptive behaviors and reducing the risk of psychopathological outcomes [62,63]. This progression is supported by steadily increasing activation in related brain regions during neurodevelopment [64,65]. Notably, early adolescence is a critical period during which the neurodevelopment is particularly vulnerable to bullying [66] and highly sensitive to acceptance and rejection, especially through social media [67]. Therefore, it is important to examine the specific impacts of cyberbullying victimization on the adolescent brain using a longitudinal design. Building on the longitudinal associations, the temporal mechanisms of cyberbullying victimization and mental health problems, as well as the mediating role of biological factors between them, can be more clearly elucidated.

### This Study

Using data from a large-scale longitudinal study in the United States—the Adolescent Brain Cognitive Development (ABCD) study [68]—this study aimed to explore the longitudinal associations among cyberbullying victimization, inhibitory control, brain activation during error processing, and mental health problems among adolescents. First, we explored how inhibitory control and ROIs’ activation during error processing at T2, both measured using the stop signal task (SST), were influenced by cyberbullying victimization at T1. We broadly hypothesized that cyberbullying victimization at T1 predicted impaired inhibitory control and altered brain activation in the error-monitoring regions at T2. Second, we examined whether the cyberbullying victimization at T1 significantly predicted the mental health problems at T2. Based on the existing evidence, we hypothesized that individuals who were victims of cyberbullying at T1 would exhibit more internalizing and externalizing problems at T2 as compared with nonvictims at T1. Finally, we hypothesized that alterations in brain activity during error processing might mediate the relationship between cyberbullying victimization and mental health problems.

## Methods

### Study Design and Participants

This retrospective cohort study uses data from the ABCD study, which is an ongoing and 10-year longitudinal study of neurocognitive development in adolescents. To form a nationally representative sample, the ABCD study used school-based probability sampling to recruit its baseline cohort between 2016 and 2018. With school selection informed by gender, race/ethnicity, socioeconomic status, and urbanicity, it enrolled adolescents aged 9-10 years from 21 sites across the United States to mitigate selection bias [69]. As data pertaining to cyberbullying are available from the 2-year follow-up and imaging data are collected every other year, the data used in this study were obtained from the 2-year follow-up (T1) and 4-year follow-up (T2) of the ABCD Study Data Release 5.1. Participants were excluded if (1) they had missing data pertaining to cyberbullying and mental health problems, (2) they had missing data pertaining to fMRI of SST, (3) their imaging data were recommended for exclusion by the ABCD team, or (4) their behavioral performance of SST was recommended for exclusion by the ABCD team. For more details regarding the exclusion of imaging data and behavioral performance of SST, please refer to the ABCD Human Subjects Study (2024) [70].

The flowchart of participant exclusion is detailed in Figure S1 in Multimedia Appendix 1 [71-75], with most of the missing data due to the loss of mental health and brain imaging data. The Little’s MCAR test suggested that the data were missing at random (*χ*^2^_24_= 31.07, *P*=.15) [76]. Therefore, participants who did not meet the criteria were deleted from the present analysis, and the final sample consisted of 1186 participants (mean age of 11.96 years; 551/1186, 46.5% female; Table 1 provides demographic information, and Table S1 in Multimedia Appendix 1 [71-75] provides comparison of included and excluded participants' characteristics). The adequacy of this sample size was evaluated using Monte Carlo simulations [77]: for the linear mixed models (LMMs) examining longitudinal associations between cyberbullying victimization, inhibitory control, brain activation during error processing, and mental health problems, these models provided 99.26% (95% CI 99.00-100.00) statistical power to detect effect size β=0.2 at α of .05. This study was reported in accordance with the STROBE (Strengthening the Reporting of Observational Studies in Epidemiology) statement [78], and the completed checklist is provided in Multimedia Appendix 2 [71-75].

**Table 1:** Descriptive statistics for demographic characteristics at T1 in the Adolescent Brain Cognitive Development study.

Characteristics		Cyberbullying victims (*n*=61)	No cyberbullying experience (*n*=1125)	*t* test (*df*)		Chi-square (*df*)		*P* value	
Age (years), mean (SD)		11.96 (0.58)	11.96 (0.64)	0.07 (1184)		—^a^		.95	
Sex, n (%)					—		0.13 (1)		.72
	Female	27 (44.3)	524 (46.6)						
	Male	34 (55.7)	601 (53.4)						
Race and ethnicity, n (%)					—		2.94 (4)		.57
	White	41 (67.2)	661 (58.8)						
	Black	4 (6.6)	100 (8.9)						
	Hispanic	9 (14.8)	219 (19.5)						
	Asian	0 (0.0)	22 (2.0)						
	Other	7 (11.5)	123 (10.9)						
Family income (US $), n (%)					—		0.48 (3)		.92
	Less than 50,000	13 (21.3)	221 (19.6)						
	50,000 through 99,999	19 (31.1)	324 (28.8)						
	100,000 and greater	25 (41.0)	512 (45.5)						
	Refused to answer/do not know	4 (6.6)	68 (6.0)						
Parents’ highest education, n (%)					—		1.36 (3)		.72
	High school education or less	8 (13.1)	143 (12.7)						
	Some college	9 (14.8)	184 (16.4)						
	Associate or Bachelor Degree	31 (50.8)	496 (44.1)						
	Post-Graduate Degree	13 (21.3)	302 (26.8)						
Offline victimization, mean (SD)		12.45 (8.81)	12.40 (4.00)	0.10 (1184)		—		.92	
Adverse childhood experiences, mean (SD)		1.47 (1.01)	1.44 (1.15)	0.21 (1184)		—		.83	
Family conflict, mean (SD)		1.91 (1.80)	1.89 (1.20)	0.09 (1184)		—		.93	
Social media use, hours, mean (SD)		0.47 (0.79)	0.51 (1.25)	0.27 (1184)		—		.79	

^a^Not applicable.

### Ethical Considerations

In accordance with the Declaration of Helsinki, the ABCD study’s procedures were reviewed, exempted, and approved by the Centralized Institutional Review Board (IRB) at the University of California, San Diego (IRB# 160091), as well as each individual site’s IRB. Informed consent was obtained from all participants and their legal guardians for both primary data collection and secondary analyses. The IRB confirmed that no additional consent was required for the secondary use of deidentified data. To ensure privacy and confidentiality, all data were deidentified and anonymized before analysis, with no personally identifiable information retained or disclosed. Participants and their families received financial compensation for their time, typically including US $200 for the parent or guardian, US $100 in gifts and gift cards for the child, and coverage for travel costs and sibling childcare. Images and Multimedia Appendix 1 [71-75] ensure participant anonymity, with no identifiable information included.

### Questionnaire Measures

#### Cyberbullying Victimization

A modified version of the cyberbullying scale [79] was used to investigate whether adolescents were victims of cyberbullying. To report cyberbullying victimization, participants responded to the question, “In the past 12 months, have you been cyberbullied where someone intentionally tried to hurt you or be mean to you online, in texts or group texts, or on social media (like Instagram or Snapchat)?”. Participants answered either “yes” or “no,” and those who responded “yes” were coded as experiencing cyberbullying victimization [80,81].

#### Mental Health Problems

The mental health problems of adolescents were assessed using the parent-reported Child Behavior Checklist [82]. On a 3-point scale (0=not true to 2=very true or very often), parents reported on the adolescent’s internalizing (T1: Cronbach α=0.88; T2: Cronbach α=0.87) and externalizing problems (Cronbach α=0.90; T2: Cronbach α=0.88). The internalizing score is the sum of all items related to “anxious depressive symptoms (12 items; eg, Fears mistakes)” and “withdrawn depressive symptoms (8 items; eg, Lacks energy),” while the externalizing score is the sum of all items related to “rule-breaking behaviors (13 items; eg, Breaks rules)” and “aggressive behaviors (17 items; eg, Gets in fights).” The raw total score on each problem dimension was converted to norm-referenced *T*-score (range with a mean of 50 and SD of 10), with a higher number indicating greater symptom severity.

### SST

The SST [83] assesses participants’ behavioral response inhibition through 300 “Go” trials and 60 “Stop” trials. During frequent “Go” trials, participants are required to press a button promptly and correctly upon seeing an arrow indicating left or right. In contrast, on “Stop” trials, participants are required to refrain from pressing the button upon seeing a vertical arrow. Both the correct “Stop” rate and stop signal reaction time (a specific calculation method is provided in Multimedia Appendix 1 [71-75]) are indicators of inhibitory control ability. Furthermore, the stop signal delay, defined as the interval between Go and Stop trials, is calibrated to maintain response inhibition accuracy at approximately 50%. If a trial is successfully inhibited, the stop signal delay is increased by 50 milliseconds, whereas it is decreased by 50 milliseconds following unsuccessful inhibition. As SST ensures nearly 50% inhibition of failure, it is optimally suited for testing brain activation during error detection. For more details regarding SST design, please refer to Casey et al [68].

### fMRI Data Acquisition and Preprocessing

The ABCD study used a harmonized neuroimaging protocol across 21 sites, using three 3T scanner platforms: Siemens Prisma (Siemens Healthineers), GE 750 (GE Healthcare), and Philips (Philips Healthcare). The ABCD’s Data Analysis, Informatics, and Resource Core was responsible for the processing of all scan data, ensuring both high quality and uniform standards across participating sites. Corrections were made for head motion, B0, nonlinearity distortions, and between-scan motion for each participant [84-86]. The ROIs’ values were derived from the average time courses of cortical regions, which were defined by FreeSurfer’s anatomical parcellations and Desikan-Killiany Atlas [87]. General linear models in AFNI’s 3dDeconvolve (Cox, 1996) were used to model task-related activation strength. Mean beta coefficients and standard errors for each ROI’s time series were calculated. For more details regarding fMRI preprocessing techniques and the analysis of task-based fMRI data, please refer to Casey et al [68] and Hagler et al [88].

**Figure 1:**
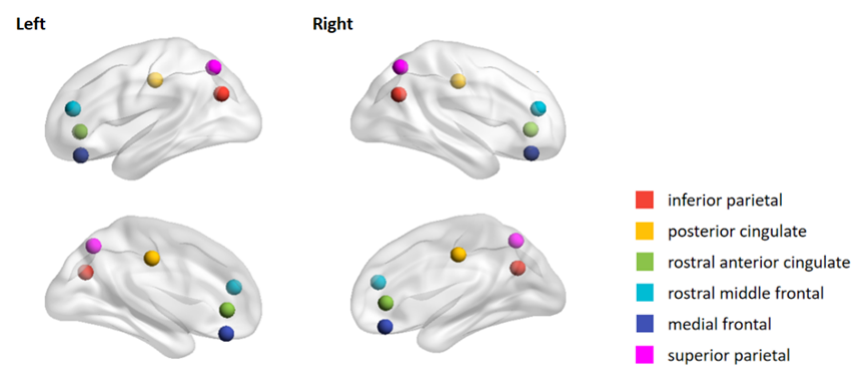
Regions of interest for failed inhibition analysis in the Adolescent Brain Cognitive Development study. The regions of interest included medial frontal, rostral middle frontal, rostral anterior cingulate, and posterior cingulate cortices. The brain activation during error processing was reflected by the mean beta weights for failed stop contrasts (ie, incorrect stop contrasted with correct go and incorrect stop contrasted with correct stop). All *P* values were adjusted for multiple comparisons using the false discovery rate method, and *P*<.05 indicated significance.

In this study, mean beta weights for failed stop contrasts (ie, incorrect stop contrasted with correct go and incorrect stop contrasted with correct stop) were used to measure brain activation during error processing [68]. Based on previous studies, we selected the cortical ROIs (Figure 1) most likely recruited by inhibitory control and error processing [43,44,53]: medial frontal, rostral middle frontal, rostral anterior cingulate, and posterior cingulate cortices. The superior and inferior parietal regions, which are closely related to attention, sensation, and adaptive behaviors [45,89], were also examined. The beta weights for each ROI in the right and left hemispheres were used in subsequent analyses.

### Covariates

Potential confounders include adolescents’ age, biological sex, race/ethnicity, family income, parents’ highest educational attainment, offline victimization, ACEs, family conflict, and social media use at T1. Adolescents’ biological sex was coded into 1=male and 2=female. Race was coded into 5 categorical variables, including 1=White, 2=Black, 3=Hispanic, 4=Asian, and 5=other races. Family income was categorized into 3 brackets, ranging from 1 (less than US $50,000) to 3 (US $100,000 and greater). Parents’ educational attainment was the highest educational degree in the family, ranging from 1 (less than a high school diploma) to 4 (postgraduate degree). Other covariates remained in the continuous format to be controlled (scoring details are provided in Multimedia Appendix 1 [71-75]).

### Statistical Analyses

All data analyses were conducted on R (version 4.4.1; R Development R Core Team, 2024). LMMs in the lme4 package [90] were used to explore the retrospective longitudinal association between cyberbullying victimization, inhibitory control, brain activation during error processing, and mental health problems. First, mental health outcomes (ie, internalizing and externalizing problems) at T2 served as the dependent variables, with whether cyberbullying victimization at T1 as the independent variable, which was included as a fixed effect in the model. Research sites and family id were treated as random effects to reflect the nested structure of the data. Second, the performance on inhibitory control (ie, correct “Stop” rate and stop signal reaction time from the SST) and the ROIs’ activation during error processing (ie, beta weight of failed stop from the SST) at T2 were used as dependent variables, while cyberbullying victimization at T1 remained the independent variable. MRI scanner type was added as a random effect in these brain measurement models.

**Figure 2:**
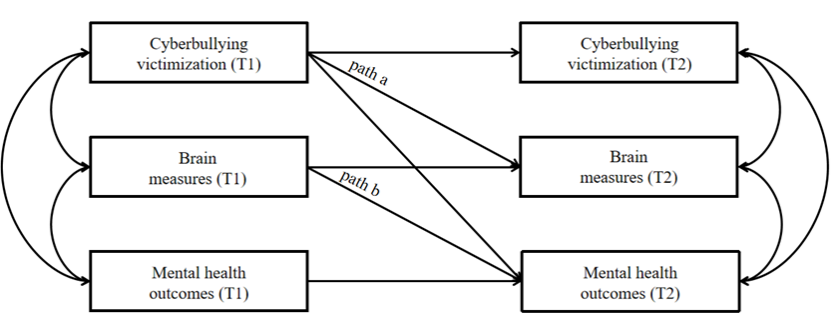
Conceptual half-longitudinal mediation model examining neural mechanisms linking cyberbullying victimization to mental health outcomes in US adolescents. For all analyses, participants’ age, biological sex, race/ethnicity, family income, parents’ highest educational attainment, offline victimization, adverse childhood experiences, family conflict, and social media use were included as covariates. Path a (T1 victimization → T2 brain measures) additionally covaried mean framewise displacement during the fMRI scan and the T1 activation level of regions of interest, while path b (T1 brain measures → T2 mental health) added T1 level of mental health to the control.

Further analyses followed up any significant findings by examining whether cyberbullying victimization at T1 was associated with mental health outcomes at T2 via altered brain activation patterns during error processing. According to Cole and Maxwell’s recommendation [91] regarding 2 waves of longitudinal studies, a half-longitudinal mediation model was established to explore the mediating role of brain activation during error processing (Figure 2). Specifically, we calculated the regression coefficient from T1 cyberbullying victimization to T2 brain measures (path a) and the regression coefficient from T1 brain measures to T2 mental health outcomes (path b). The significance of the mediating effect (path a × b) was calculated using bootstrap analysis with 5000 resamples to generate bias-corrected CI. When the 95% CI does not contain 0, then the mediating effect of brain activation is statistically significant.

Adolescents’ biological sex, age, race/ethnicity, family income, and highest parental education attainment were included as demographic covariates in all analyses. In the analyses predicting brain activation of SST, we additionally covaried mean framewise displacement and its quadratic effects during the fMRI scan [92]. Furthermore, to control for the developmental effect of the dependent variable in each LMM, we separately covaried T1 level of mental health, inhibitory control, and activation of ROIs during error processing in the corresponding model [71,93]. For example, the T1 level of externalizing problems was included as a covariate in the LMM exploring the link between whether cyberbullying victimization at T1 and T2 levels of externalizing problems. Finally, we used the inverse probability weighting method [94] in the survey package [95] to test the robustness of the results from all LLMs and mediation models. False discovery rate (FDR) was applied to correct for multiple comparisons, and *P*_FDR_<.05 was considered to reach the significant level.

## Results

### Descriptive Statistics for Demographic Characteristics

Descriptive statistics for demographic characteristics at T1 are presented in Table 1. Independent samples *t* tests and chi-square test indicated that no significant difference was found between the cyberbullying victims and participants with no cyberbullying experiences in age, offline victimization, ACEs, family conflict, and social media use, t_1184_< 0.27, *P*>.79, sex, race/ethnicity, family income, or parents’ highest educational attainment, *χ*^2^_4_< 2.94, *P*>.57.

### Associations Between Cyberbullying at T1 and ROIs’ Activation During Error Processing at T2

The associations between cyberbullying victimization at T1 and ROIs’ activation during error processing at T2 are shown in Table 2. For the contrast of incorrect stop versus correct go, cyberbullying victimization at T1 was significantly associated with higher activation of bilateral superior parietal gyri (left: β=0.36, 95% CI 0.10-0.61, SE 0.01, *P*_FDR_=.04; right: β=0.34, 95% CI 0.08-0.59, SE 0.01, *P*_FDR_=.04), right inferior parietal gyrus (β=0.32, 95% CI 0.07-0.57, SE 0.01, *P*_FDR_=.04), and right posterior cingulate cortex (β=0.34, 95% CI 0.09-0.60, SE 0.01, *P*_FDR_=.04) at T2. For the contrast of incorrect stop versus correct stop, significant heightened activation was also observed in the bilateral superior parietal gyri (left: β=0.32, 95% CI 0.08-0.52, SE 0.01, *P*_FDR_=.04; right: β=0.30, 95% CI 0.05-0.48, SE 0.01, *P*_FDR_=.04), bilateral inferior parietal gyri (left: β=0.30, 95% CI 0.06-0.50, SE 0.01, *P*_FDR_=.04; right: β=0.30, 95% CI 0.04-0.47, SE 0.01, *P*_FDR_=.04), and right posterior cingulate cortex (β=0.34, 95% CI 0.10-0.60, SE 0.01, *P*_FDR_=.04). No other significant ROIs’ activated alteration during error processing at T2 were predicted by cyberbullying victimization at T1 after correction for multiple comparisons.

**Table 2:** Associations between cyberbullying victimization at T1 and cortical regions of interest activation during error processing at T2 in the Adolescent Brain Cognitive Development study.

		Left hemisphere						Right hemisphere				
		β^a^	95% CI	SE^b^	*P* value^c^	*P*_FDR_ value^c^	β		95% CI	SE	*P* value^c^	*P*_FDR_ value^c^
Incorrect stop contrasted with correct go												
	Superior parietal	0.36	0.10 to 0.61	0.01	.005^d^	.04^d^	0.34		0.08 to 0.59	0.01	.009^d^	.04^d^
	Inferior parietal	0.26	0.00 to 0.51	0.01	.047^d^	.09	0.32		0.07 to 0.57	0.01	.01^d^	.04^d^
	Medial frontal	0.22	–0.03 to 0.48	0.03	.08	.14	0.20		–0.06 to 0.45	0.03	.13	.20
	Rostral middle frontal	0.15	–0.10 to 0.41	0.02	.24	.28	0.17		–0.09 to 0.42	0.02	.20	.27
	Rostral anterior cingulate	–0.01	–0.27 to 0.24	0.02	.91	.91	–0.03		–0.29 to 0.22	0.02	.81	.88
	Posterior cingulate	0.27	0.01 to 0.53	0.01	.04^d^	.09	0.34		0.09 to 0.60	0.01	.008^d^	.04^d^
Incorrect stop contrasted with correct stop												
	Superior parietal	0.32	0.08 to 0.52	0.01	.006^d^	.04^d^	0.30		0.05 to 0.48	0.01	.01^d^	.04^d^
	Inferior parietal	0.30	0.06 to 0.50	0.01	.01^d^	.04^d^	0.30		0.04 to 0.47	0.01	.02^d^	.04^d^
	Medial frontal	0.17	–0.08 to 0.38	0.04	.10	.17	0.22		–0.06 to 0.45	0.03	.11	.17
	Rostral middle frontal	–0.03	–0.28 to 0.23	0.02	.83	.83	0.05		–0.21 to 0.31	0.02	.70	.76
	Rostral anterior cingulate	–0.06	–0.31 to 0.20	0.02	.66	.76	–0.02		–0.27 to 0.21	0.02	.63	.76
	Posterior cingulate	0.28	0.04 to 0.58	0.01	.04^d^	.08	0.34		0.10 to 0.60	0.01	.006^d^	.04^d^

^a^β: standardized coefficient.

^b^SE of β*.*

^c^All *P* values were adjusted for multiple comparisons using the false discovery rate (FDR) method.

^d^*P*<.05.

### Associations Between Cyberbullying at T1 and Mental Health Outcomes/Inhibitory Control at T2

Descriptive statistics for mental health outcomes and inhibitory control at T1 and T2 are presented in Table 3. The LMMs revealed that cyberbullying victimization at T1 significantly predicted increased externalizing problems at T2 (β=0.25, 95% CI 0.06-0.45, SE 0.87, *P*_FDR_=.02). In contrast, internalizing problems and inhibitory control performance at T2 were not significantly associated with cyberbullying victimization at T1 (Table 4).

**Table 3:** Descriptive statistics for mental health outcomes and stop signal task (SST) behavioral performance at T1 and T2 in the Adolescent Brain Cognitive Development study.

		T1		T2	
		Cyberbullying victims (n=61)	No cyberbullying experience (n=1125)	Cyberbullying victims (n=61)	No cyberbullying experience (n=1125)
Mental health outcomes, mean (SD)					
	Internalizing problems	49.39 (9.33)	47.65 (10.34)	48.85 (8.76)	47.85 (10.72)
	Externalizing problems	46.69 (8.92)	44.17 (9.10)	46.97 (8.90)	43.08 (8.77)
SST behavioral performance, mean (SD)					
	Correct stop rate (%)	50.10 (6.60)	50.90 (5.40)	48.80 (6.90)	49.20 (6.30)
	Stop signal reaction time (ms)	284.41 (58.00)	276.84 (57.24)	264.49 (56.36)	261.73 (50.77)

**Table 4:** Associations between cyberbullying victimization at T1 and mental health outcomes/inhibitory control at T2 in the Adolescent Brain Cognitive Development study.

		β^a^	95% CI	SE^b^	*P* value^c^	*P*_FDR_ value^c^
Mental health outcomes						
	Internalizing problems	–0.01	–0.20 to 0.19	1.06	.99	.99
	Externalizing problems	0.25	0.06 to 0.45	0.87	.01	.02^d^
SST^e^ behavioral performance						
	Correct stop rate (%)	–0.02	–0.26 to 0.21	0.01	.85	.85
	Stop signal reaction time	–0.07	–0.27 to 0.13	5.14	.49	.85

^a^β: standardized coefficient.

^b^SE of β*.*

^c^All *P* values were adjusted for multiple comparisons using the false discovery rate (FDR) method.

^d^*P*<.05.

^e^SST: stop signal task.

### Mediation Analyses

As shown in Table 5, the mediation effects of parietal gyri or posterior cingulate cortex activated alteration during error processing between cyberbullying victimization at T1 and externalizing problems at T2 did not reach a significant level after correction for multiple comparisons (*P*_FDR_s>.74; specific path coefficients are presented in Table S2 in Multimedia Appendix 1 [71-75]).

**Table 5:** Mediation effects of brain activation during error processing between cyberbullying victimization at T1 and externalizing problems at T2 in the Adolescent Brain Cognitive Development study.

			Left hemisphere									Right hemisphere						
			Indirect effect		95% CI		*P* value^a^		*P*_FDR_ value^a^		Indirect effect			95% CI		*P* value^a^		*P*_FDR_ value^a^
Incorrect stop contrasted with correct go																		
	Superior parietal	0.004		–0.003 to 0.006		.66		>.99		0.001			–0.005 to 0.005		>.99		>.99	
	Inferior parietal	0.000		–0.003 to 0.004		.87		>.99		–0.004			–0.007 to 0.002		.47		>.99	
	Posterior cingulate	–0.005		–0.006 to 0.004		.62		>.99		–0.006			–0.007 to 0.005		.65		>.99	
Incorrect stop contrasted with correct stop																		
	Superior parietal	0.003		–0.002 to 0.004		.74		.82		0.003			–0.001 to 0.005		.64		.82	
	Inferior parietal	0.003		–0.002 to 0.004		.82		.82		0.006			–0.001 to 0.008		.74		.82	
	Posterior cingulate	0.005		–0.004 to 0.006		.54		.82		0.006			–0.001 to 0.008		.44		.82	

^a^All *P* values were adjusted for multiple comparisons using the false discovery rate (FDR) method.

### Inverse Probability Weighting Analysis

After inverse probability weighting, the results remained consistent with the primary analyses (Tables S3-S6 in Multimedia Appendix 1 [71-75]). Specifically, cyberbullying victimization at T1 was significantly associated with higher activation in the bilateral superior parietal gyri, inferior parietal gyri, and right posterior cingulate cortex at T2 (Table S3 in Multimedia Appendix 1 [71-75]). Behaviorally, while it significantly predicted increased externalizing problems, its associations with internalizing problems and inhibitory control performance still did not reach a significant level (Table S4 in Multimedia Appendix 1 [71-75]). Moreover, the mediation effects of activation in the parietal gyri or posterior cingulate cortex during error processing between cyberbullying victimization and externalizing problems were also nonsignificant (Tables S5 and S6 in Multimedia Appendix 1 [71-75]).

## Discussion

### Principal Findings

This retrospective cohort study explored the longitudinal association between cyberbullying victimization, inhibitory control, brain activation during error processing, and mental health problems among American adolescents. Adolescents who experienced cyberbullying victimization demonstrated heightened activation in the parietal gyri and posterior cingulate cortices during error processing. Additionally, these victims showed more subsequent externalizing rather than internalizing problems. However, the mediating effects of brain-activated alteration on the link between cyberbullying victimization and mental health outcomes did not reach significant levels.

### Brain Activation During Error Processing

Our study showed that cyberbullying victimization significantly contributed to higher activation in parietal regions and posterior cingulate cortices during error processing, which may reflect their high sensitivity and vigilance to adversity, thus avoiding potential injury [54]. The parietal cortex plays a critical role in an individual’s attention regulation, goal orientation, and motor coordination [45,88]. Increased activation of the parietal cortex may not only make cyberbullying victims more vigilant and sensitive to adversity, but also underpin their subsequent behavioral adjustments to distance themselves from potential injury. Victims of cyberbullying experienced web-based, anonymous, and spatially/temporally unlimited harm (eg, verbal scold and satirize) in a web-based environment [10,96], potentially leading them to distance themselves from various harm in any real-life situations. This generalized avoidance tendency could be supported by the parietal gyri, given their involvement in relevant cognitive functions.

The posterior cingulate cortex, a core structure in the default mode network, is closely associated with an individual’s self-reflection, attention shift, and emotion regulation [97,98]. However, its interpretation in our findings requires caution, as this region typically deactivates during goal-directed tasks in typical populations [99]. Therefore, the observed higher activation among victims may not reflect an absolute increase above a resting baseline, but rather a reduction in the deactivation. This pattern could signify either a failure to suppress self-referential processing, potentially due to intrusive thoughts related to victimization experiences, or the active recruitment of cognitive resources for heightened internal monitoring and emotion regulation to stabilize the internal state during adversity [100]. It is also worth noting that the significant change in activation of the inferior parietal and posterior cingulate cortices was only observed in the right hemisphere. Combined with the fact that no significant difference in frontal and anterior cingulate activation was found between cyberbully victims and nonvictims, it seems that the effects of cyberbullying partially lead to an increased cognitive load, but do not widely damage the error-monitoring circuits and cognitive control network.

From the perspective of latent vulnerability theory [101,102], a nonsignificant change in the anterior cingulate cortex but significantly higher activation in posterior cingulate and parietal cortices could be viewed as adaptive responses and adjustments, rather than mere damage after cyberbullying victimization. Furthermore, these changes could serve as generalized neural impacts of this experience, as this study only used category variables to define cyberbullying but did not consider its intensity. However, given that this form of victimization has been shown to elicit higher cortisol secretion levels and greater perceived stress in adolescents [58], which could disrupt the hypothalamic-pituitary-adrenal axis feedback [103], further studies are warranted to further explore the relationship between characteristic neural alterations and the intensity of cyberbullying victimization.

### Externalizing Problems

The relationship between cyberbullying victimization and externalizing problems was consistent with previous studies [13-15]. Yet the mediating effects of brain over-activation during error processing between cyberbullying victimization and externalizing problems did not reach a significant level. As mentioned above, the higher activation of parietal and posterior cingulate cortices in SST may serve as adaptive adjustments, rather than severe neural circuit damage. Moreover, dealing with real-life “errors” may generally require more cognitive resources and neural load than processing failed inhibition in SST. In other words, the standardized stimuli and the monotonous failed inhibition in SST may not adequately elicit the actual neural changes of cyberbullying victimization. Consequently, despite certain differences in brain activation measured by the SST between cyberbullying victims and nonvictims, such differences could not sufficiently mediate the relationship between cyberbullying victimization and externalizing problems.

The sustained performance of inhibitory control in cyberbullying victims also provides a potential explanation for the insufficient mediated effect of brain-activated alteration. Previous studies suggested that poor inhibitory control is a predictor of externalizing problems [104,105]. However, no significant predictive difference was found in inhibitory control performance between cyberbullying victims and nonvictims of this study, which aligned with Lim et al [53] and Carrion et al [106]. Therefore, we speculate that the higher neurological load in the parietal gyri and posterior cingulate cortices did not lead to more failed inhibition in SST (ie, decompensatory effect), but rather maintained the performance of inhibitory control (ie, compensatory effect) with heightened attention and self-reflection, resulting in no evidence for a significant mediation effect for externalizing problems. In general, both the potential lack of representation and the protective bias role of altered brain activation measured by SST may contribute to this study’s failure to indicate the significant mediated brain effects.

Based on existing studies, the link between cyberbullying victimization and externalizing problems may be effectively explained by other mechanisms. One potential mechanism is that the experience of cyberbullying elicited the victim’s aggressive tendencies (eg, hostility and retaliatory motivation) [14,107,108]. For example, Tong et al [14] engaged 464 rural adolescents to examine the association between cyberbullying victimization and malevolent creativity. The results revealed that victims mediated increased hostile attribution leading to serious malevolent creativity. Another potential mechanism is that cyberbullying victimization led to the adoption of externalizing behaviors through imitation [107,109]. Specifically, according to the general learning model [110], we speculate that cyberbullying is a situational factor that influences victims’ observational learning processes. These influences manifested in internal states, including perception and emotion, and subsequently contributed to externalizing problems. For instance, victims of cyberbullying perceived the characteristics of the bullying conducted in the web-based environment (eg, anonymity and no strength differential). They may develop a positive attitude towards such characteristics, thereby engaging in cyberbullying [107,109].

Overall, this study revalidated the longitudinal association between cyberbullying and externalizing problems but failed to indicate the significant neural mediation measured by SST. More studies are needed in the future to comprehensively explore other potential mediated mechanisms.

### Internalizing Problems

Findings in previous studies regarding whether the cyberbullying victimization could predict internalizing problems (eg, depression and anxiety) are controversial [9,10,17,18]. Similarly with Chu et al [17] and Frison et al [18], this study found that cyberbullying victimization did not significantly predict internalizing problems. One potential explanation is that individuals have the ability to regulate their emotions, which might mitigate the internalized impacts of cyberbullying [111,112]. Since the emotional regulation is closely linked to the activation of the posterior cingulate cortex and the performance of inhibitory control [97,100], we speculate that heightened activation of the posterior cingulate cortex during error processing may reflect cyberbullying victims’ increased load of emotional regulation to cope with adversity. Such increased load may also serve as the neural compensation and protection, through which cyberbullying victimization could not directly and severely disrupt the individual’s internal state.

Another potential explanation is the different measurement methods used for cyberbullying and emotions. Specifically, cyberbullying is a trait measure (ie, assessing the frequency of cyberbullying within a certain time frame by self-report) [113], and emotions are conceptualized as state processes (ie, measurement dependent on current arousal) [114]. The mismatch between these measurement methods leads to an inability to sensitively capture the immediate impact of cyberbullying on emotions. Therefore, we speculate that the significant longitudinal effect of cyberbullying on internalizing problems only emerges when there is sufficient emotional destructiveness and prolonged duration of victimization, which aligns with the hypothesis proposed by Frison et al [18]. Given that this study used a categorical variable to define cyberbullying, which may be more reflective of general victimization, future studies should explore whether there is a longitudinal association between the intensity and frequency of cyberbullying and the severity of neural damage, as well as internalizing problems [21].

### Theoretical and Practical Implications

For theoretical implications, our findings promote the integration of the error detection theory [35,36] and psychopathological outcomes. The potential neurocompensatory response observed in the posterior cingulate and parietal cortices during failed inhibition could provide a novel account for the stress process model [22], illustrating how chronic social stress may manifest as heightened neural sensitivity to errors while maintaining behavioral performance. Similarly, these neural findings offer a mechanistic elaboration of the general aggression model [23,115] for externalizing problems, specifying how victimization might strain cognitive control resources without immediate behavioral manifestations. For practical implications, the significant longitudinal association with externalizing problems highlights the importance of incorporating cyberbullying victimization screening into mental health assessments at an early stage. Furthermore, the neural signature identified here might inform the development of targeted interventions, such as cognitive training protocols designed to reinforce compensatory processes, which hold potential for enhancing emotion regulation and mitigating the risk of externalizing behaviors in affected youth [116,117].

### Limitations and Future Directions

This study has a few limitations. The first limitation stems from data availability in the ABCD Study Release 5.1. The analytical sample size was reduced primarily due to the partial availability of the 4-year follow-up (T2) data. This attrition likely contributed to the relatively low prevalence of cyberbullying victims (≈5%), which may limit statistical power to detect smaller effects. Furthermore, although our analysis did not reveal systematic differences in baseline characteristics between included and excluded participants, the potential selection bias (eg, the unexpected similarity in offline victimization levels between victims and nonvictims) should be considered when interpreting the results and their generalizability.

Second, the analysis was constrained to only 2 time points (T1 and T2) available in this data release. This limits the representation of developmental trajectories and the more rigorous longitudinal mediation analysis (eg, a T1→T2→T3 pathway). Future studies should incorporate larger samples of affected individuals and more waves of neuroimaging data across development to examine how cyberbullying victimization influences mental health via neural changes over time.

Third, cyberbullying was analyzed as a categorical variable (victims vs nonvictims) in this study. This approach was adopted because the item measuring frequency/intensity had a high rate of missing data in our analytical sample, which precludes examining the impact of victimization severity on mental health problems, especially internalizing problems. Moreover, this study only focused on externalizing problems, internalizing problems, and brain activation during error processing. Cyberbullying may be significantly associated with other mental/physical health problems (eg, eating disorder, suicide, and sleep disturbance) [79,118,119] as well as social media use [67,71], it is necessary to comprehensively examine the longitudinal impacts of cyberbullying on a variety of health problems and brain states (eg, morphology, resting-state connectivity, and other dynamic states).

### Conclusions

Drawing on the ABCD study, this fMRI investigation advances prior cross-sectional or behavioral research by adopting a longitudinal neurobiological perspective. It reveals that cyberbullying victimization predicts significantly heightened activation in parietal and posterior cingulate regions during error processing, alongside increased externalizing problems over time. The absence of significant deficits in inhibitory control and mediation effects suggests that these neural impacts may be better characterized by a state of heightened sensitivity and compensatory engagement after victimization rather than direct impairment. In general, these findings identify the error-processing network as a candidate neural system for future study and motivate continued exploration of other neural pathways linking cyberbullying victimization to mental health, with the ultimate aim of mitigating negative consequences.

## Appendix

Multimedia Appendix 1 Additional material.

Multimedia Appendix 2 STROBE checklist.

## Abbreviations

ABCD: Adolescent Brain Cognitive Development
ACE: adverse childhood experience
FDR: false discovery rate
fMRI: functional magnetic resonance imaging
IRB: Institutional Review Board
LMM: linear mixed model
ROI: regions of interest
SST: stop signal task
STROBE: Strengthening the Reporting of Observational Studies in Epidemiology

## Data Availability

Data were obtained from the 2-year follow-up and 4-year follow-up of the Adolescent Brain Cognitive Development (ABCD) Study (data release 5.1). The data that support the findings of this study are available at the NIMH Data Archive upon data access request.
