# Supplementary material for: Associations Among Cyberbullying Victimization, Inhibitory Control, Neural Activation of Error Processing, and Mental Health Problems in Adolescents: Neuroimaging, Retrospective Longitudinal Cohort Study Using the Adolescent Brain Cognitive Development Data
Source: J Med Internet Res. 2026 Feb 18;28:e75126. doi: 10.2196/75126 (PMC12961395; doi:10.2196/75126)
Supplement: Multimedia Appendix 1 [file jmir_v28i1e75126_app1.docx]

**Supplementary materials**

**Associations between cyberbullying victimization, neural activation of error processing, and mental health problems in adolescents: A longitudinal study using the Adolescent Brain Cognitive Development data**

**Details** about Stop Signal Reaction Time (SSRT), offline victimization, adverse childhood experiences (ACEs), family conflict, and social media use.

**Figure S1.** Participant exclusion flowchart for the ABCD study analyzing the longitudinal association between cyberbullying victimization, brain activation during error processing, and mental health problems.

**Table S1.** Characteristics of included and excluded participants at T1 in the ABCD study.

**Table S2.** Path coefficients in the longitudinal mediation analysis of cyberbullying victimization, brain activation during error processing, and externalizing problems in the ABCD Study.

**Table S3.** Associations between cyberbullying victimization at T1 and cortical ROIs activation during error processing at T2 in the ABCD study after inverse probability weighting.

**Table S4.** Associations between cyberbullying victimization at T1 and mental health outcomes/inhibitory control at T2 in the ABCD study after inverse probability weighting.

**Table S5.** Mediation effects of brain activation during error processing between cyberbullying victimization at T1 and externalizing problems at T2 in the ABCD study after inverse probability weighting.

**Table S6.** Path coefficients in the longitudinal mediation analysis of cyberbullying victimization, brain activation during error processing, and externalizing problems in the ABCD Study after inverse probability weighting.

**Stop-Signal Reaction Time (SSRT)**

In this study, SSRT was computed using the integration method [1]. This approach first calculates the probability of inhibition as the ratio of successful “Stop” trials to total “Stop” trials. The corresponding percentile in the “Go” reaction time distribution is then determined as (1 - *p*(inhibit)) × 100. Finally, SSRT is obtained by subtracting the mean Stop Signal Delay from the “Go” reaction time at this specific percentile. This method provides a robust measure of inhibitory control by utilizing the complete distribution of response times.

**Offline victimization**

The offline victimization of adolescents were assessed using the Peer Experiences Questionnaire[2]. Questions cover domains of overt (3 items; e.g., “A kid threatened to hurt or beat me up”), relational (3 items; e.g., “A kid left me out of what they were doing”), or reputational victimization (3 items; e.g., “A kid tried to damage my social reputation by spreading rumors about me”) on a 5-point scale ranging from 1 (“Never”) to 5 (“A few times a week”). The total score was calculated as the sum of all items (T1: Cronbach's α = 0.76; T2: Cronbach's α = 0.77), with higher scores reflecting more severe offline victimization.

**Adverse childhood experiences (ACEs)**

The ACEs of adolescents were assessed using items derived from the original CDC-Kaiser ACE study [3]. The measure covers eight types of adversity: physical abuse (2 items; e.g., “Shot, stabbed, or beaten brutally by a grown up in the home

”), sexual abuse (2 items; e.g., “A grown up in the home touched your child in their privates.”), household violence (5 items; e.g., “Witness the grownups in the home push, shove or hit one another”), household mental illness (2 items; e.g., “Has any blood relative of your child ever attempted or committed suicide?”), household substance abuse(1 item; i.e., “Has any blood relative of your child ever had any problems due to alcohol such as: marital separation or divorce, laid off or fired from work, arrests or DUIs; alcohol harmed their health; in an alcohol treatment program; suspended or expelled from school 2 or more times; isolated self from family, caused arguments or were drunk a lot?”), parental divorce/separation (1 item; i.e., “Divorced/separated.”), emotional neglect (1 item; i.e., “Believes in showing his/her love for me.”), and physical neglect (2 items; e.g., “How often do your parents/guardians know where you are?”). Responses were recorded as binary indicators (0 = no, 1 = yes) based on parent or children reports, and the cumulative ACE score was calculated by summing all affirmative responses, with higher scores indicating greater exposure to childhood adversity.

**Family conflict**

The family conflict of adolescents were assessed using the Conflict subscale from the Family Environment Scale [4]. On a binary response scale (0 = no, 1 = yes), parents completed nine items (e.g., Family members sometimes hit each other) to report the level of openly expressed anger, conflict, and tension among family members. The total score was calculated as the sum of all items (T1: Cronbach's α = 0.71; T2: Cronbach's α = 0.70), with higher scores reflecting more severe family conflict.

**Social media use**

To report social media use, adolescents responded to the question, “how many hours per weekday/weekend day do you spend on social media?” [5].

**Reference**

1. Verbruggen F, Aron AR, Band GP, Beste C, Bissett PG, Brockett AT, Brown JW, Chamberlain SR, Chambers CD, Colonius H, Colzato LS, Corneil BD, Coxon JP, Dupuis A, Eagle DM, Garavan H, Greenhouse I, Heathcote A, Huster RJ, Jahfari S, Kenemans JL, Leunissen I, Li C-SR, Logan GD, Matzke D, Morein-Zamir S, Murthy A, Paré M, Poldrack RA, Ridderinkhof KR, Robbins TW, Roesch M, Rubia K, Schachar RJ, Schall JD, Stock A-K, Swann NC, Thakkar KN, van der Molen MW, Vermeylen L, Vink M, Wessel JR, Whelan R, Zandbelt BB, Boehler CN. A consensus guide to capturing the ability to inhibit actions and impulsive behaviors in the stop-signal task. eLife 2019 Apr 29;8:e46323. PMID:31033438

2. Prinstein MJ, Boergers J, Vernberg EM. Overt and Relational Aggression in Adolescents: Social-Psychological Adjustment of Aggressors and Victims. J Clin Child Adolesc Psychol Routledge; 2001 Nov 1;30(4):479–491. PMID:11708236

3. Felitti VJ, Anda RF, Nordenberg D, Williamson DF, Spitz AM, Edwards V, Koss MP, Marks JS. Relationship of Childhood Abuse and Household Dysfunction to Many of the Leading Causes of Death in Adults: The Adverse Childhood Experiences (ACE) Study. Am J Prev Med Elsevier; 2019 Jun 1;56(6):774–786. PMID:31104722

4. Lanz M, Maino E. Family Environment Scale. Encycl Qual Life Well- Res Springer, Dordrecht; 2014. p. 2170–2173. doi: 10.1007/978-94-007-0753-5_999ISBN:978-94-007-0753-5

5. He X, Hu J, Yin M, Zhang W, Qiu B. Screen Media Use Affects Subcortical Structures, Resting-State Functional Connectivity, and Mental Health Problems in Early Adolescence. Brain Sci 2023 Oct 12;13(10):1452. PMID:37891820


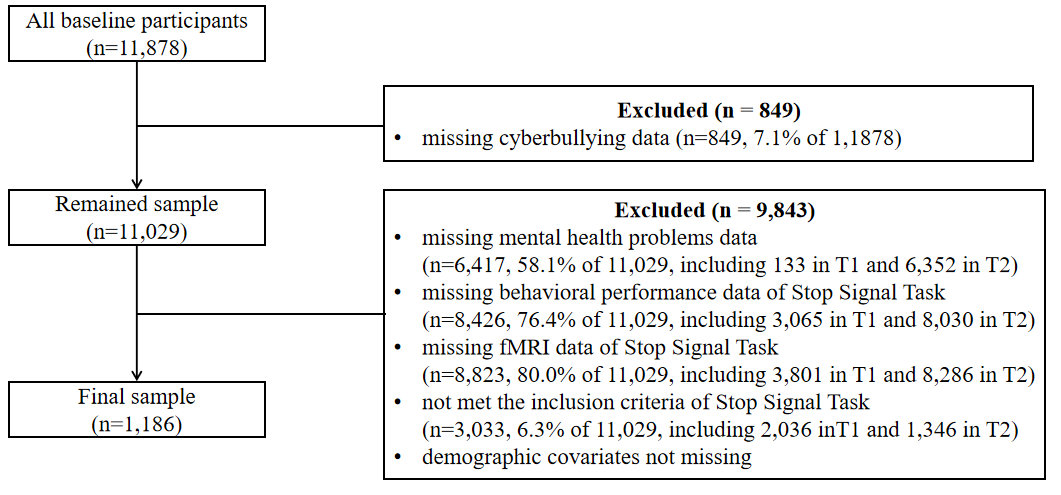
**Figure S1.** Participant exclusion flowchart for the ABCD study analyzing the longitudinal association between cyberbullying victimization, brain activation during error processing, and mental health problems.

**Table S1.** Characteristics of included and excluded participants at T1 in the ABCD study.

| Characteristics | Excluded participants  (*n* = 10,682) | Included participants  (*n* = 1,186) | *t*/*χ^2^* | *P* |
| --- | --- | --- | --- | --- |
| **Age, years, mean (*SD*)** | 12.01 (0.67) | 11.96 (0.62) | 2.45 | .014 |
| **Sex, n (%)** |  |  |  |  |
| Female | 5,129 (48.0%) | 551 (46.4%) | 1.04 | .300 |
| Male | 5,553 (52.0%) | 635 (53.6%) |  |  |
| **Race and ethnicity, n (%)** |  |  | 48.89 | <.001 |
| White | 5,471 (51.2%) | 702 (59.2%) |  |  |
| Black | 1,680 (15.7%) | 104 (8.8%) |  |  |
| Hispanic | 2,182 (20.4%) | 228 (19.2%) |  |  |
| Asian | 230 (2.2%) | 22 (1.9%) |  |  |
| Other | 1,117 (10.5%) | 130 (11.0%) |  |  |
| **Family income, n (%)** |  |  | 1.54 | .673 |
| less than USD 50,000 | 2,253 (21.1%) | 234 (19.7%) |  |  |
| USD 50,000 through USD 99,999 | 2,345 (22.0%) | 343 (28.9%) |  |  |
| USD 100,000 and greater | 4,297 (40.2%) | 537 (45.3%) |  |  |
| Refused to Answer/Don’t Know | 1,787 (16.7%) | 72 (6.1%) |  |  |
| **Parents’ highest education, n (%)** |  |  | 3.36 | .339 |
| High school education or less | 1,604 (15.0%) | 151 (12.7%) |  |  |
| Some College | 1,498 (14.0%) | 193 (16.3%) |  |  |
| Associate’s or Bachelor’s Degree | 3,981 (37.3%) | 527 (44.4%) |  |  |
| Post-Graduate Degree | 2,619 (24.5%) | 315 (26.6%) |  |  |
| **Cyberbullying victimization, n (%)** | 936 (9.6%) | 61 (5.1%) | 25.55 | <.001 |
| **Offline victimization, mean (*SD*)** | 12.36 (4.19) | 12.41 (4.08) | 0.39 | .696 |
| **Adverse childhood experiences, mean (*SD*)** | 1.46 (1.05) | 1.45 (1.12) | 0.31 | .756 |
| **Family conflict, mean (*SD*)** | 1.89 (1.90) | 1.91 (1.76) | −0.35 | .727 |
| **Social media use, hours, mean (*SD*)** | 0.56 (1.20) | 0.51 (1.18) | 1.36 | .174 |

**Table S2.** Path coefficients in the longitudinal mediation analysis of cyberbullying victimization, brain activation during error processing, and externalizing problems in the ABCD Study.

|  | Left hemisphere | | | | | | | | Right hemisphere | | | | | | | |
| --- | --- | --- | --- | --- | --- | --- | --- | --- | --- | --- | --- | --- | --- | --- | --- | --- |
|  | Path a | 95% *CI* | *P* | *P*_FDR_ | Path b | 95% *CI* | *P* | *P*_FDR_ | Path a | 95% *CI* | *P* | *P*_FDR_ | Path b | 95% *CI* | *P* | *P*_FDR_ |
| **Incorrect stop contrasted with correct go** | | | | | | | | | | | | | | | | |
| Superior parietal | 0.36 | [0.10, 0.61] | .005^a^ | .018^a^ | 0.01 | [-0.03, 0.05] | .588 | .882 | 0.34 | [0.08, 0.59] | .009^a^ | .018^a^ | 0.00 | [-0.04, 0.05] | .903 | .935 |
| Inferior parietal | 0.26 | [0.00, 0.51] | .047^a^ | .047^a^ | 0.00 | [-0.04, 0.04] | .935 | .935 | 0.32 | [0.07, 0.57] | .013^a^ | .020^a^ | -0.01 | [-0.06, 0.03] | .554 | .882 |
| Posterior cingulate | 0.27 | [0.01, 0.53] | .038^a^ | .046^a^ | -0.02 | [-0.06, 0.03] | .430 | .882 | 0.34 | [0.09, 0.60] | .008^a^ | .018^a^ | -0.02 | [-0.06, 0.02] | .393 | .882 |
| **Incorrect stop contrasted with correct stop** | | | | | | | | | | | | | | | | |
| Superior parietal | 0.32 | [0.08, 0.52] | .006^a^ | .018^a^ | 0.01 | [-0.03, 0.05] | .627 | .644 | 0.30 | [0.05, 0.48] | .011^a^ | .018^a^ | 0.01 | [-0.02, 0.05] | .422 | .644 |
| Inferior parietal | 0.30 | [0.06, 0.50] | .013^a^ | .018^a^ | 0.01 | [-0.03, 0.05] | .644 | .644 | 0.30 | [0.04, 0.47] | .015^a^ | .018^a^ | 0.02 | [-0.02, 0.06] | .436 | .644 |
| Posterior cingulate | 0.28 | [0.04, 0.58] | .038^a^ | .006^a^ | 0.02 | [-0.01, 0.06] | .213 | .639 | 0.34 | [0.10, 0.60] | .006^a^ | .018^a^ | 0.02 | [-0.01, 0.05] | .201 | .639 |

*Note*. *CI* = confidence interval. The path a is from T1 cyberbullying victimization to T2 brain activation during error processing, and the path b is from T1brain activation during error processing to T2 externalizing problems. All *P* values were adjusted for multiple comparisons using the false discovery rate (FDR) method and and a indicates *P* < .05.

**Table S3.** Associations between cyberbullying victimization at T1 and cortical ROIs activation during error processing at T2 in the ABCD study after inverse probability weighting.

|  | Left hemisphere | | | | | Right hemisphere | | | | |
| --- | --- | --- | --- | --- | --- | --- | --- | --- | --- | --- |
|  | *β* | 95% *CI* | *SE* | *P* | *P*_FDR_ | *β* | 95% *CI* | *SE* | *P* | *P*_FDR_ |
| **Incorrect stop contrasted with correct go** | | | | | | | | | | |
| Superior parietal | 0.36 | [0.11, 0.61] | 0.01 | .004^a^ | .024^a^ | 0.34 | [0.10, 0.59] | 0.01 | .006^a^ | .024^a^ |
| Inferior parietal | 0.26 | [0.01, 0.51] | 0.01 | .041^a^ | .082 | 0.33 | [0.08, 0.58] | 0.01 | .009^a^ | .027^a^ |
| Medial frontal | 0.21 | [-0.04, 0.46] | 0.03 | .106 | .181 | 0.20 | [-0.06, 0.45] | 0.03 | .134 | .201 |
| Rostral middle frontal | 0.18 | [-0.08, 0.43] | 0.02 | .172 | .206 | 0.18 | [-0.07, 0.43] | 0.02 | .159 | .206 |
| Rostral anterior cingulate | -0.01 | [-0.27, 0.24] | 0.02 | .918 | .980 | -0.00 | [-0.26, 0.25] | 0.02 | .980 | .980 |
| Posterior cingulate | 0.28 | [0.03, 0.54] | 0.01 | .028^a^ | .067 | 0.36 | [0.11, 0.61] | 0.01 | .005^a^ | .024^a^ |
| **Incorrect stop contrasted with correct stop** | | | | | | | | | | |
| Superior parietal | 0.32 | [0.10, 0.52] | 0.01 | .005^a^ | .030^a^ | 0.31 | [0.06, 0.48] | 0.01 | .010^a^ | .033^a^ |
| Inferior parietal | 0.30 | [0.08, 0.50] | 0.01 | .011^a^ | .033^a^ | 0.30 | [0.07, 0.51] | 0.01 | .014^a^ | .034^a^ |
| Medial frontal | 0.16 | [-0.10, 0.37] | 0.04 | .183 | .308 | 0.21 | [-0.08, 0.45] | 0.03 | .205 | .308 |
| Rostral middle frontal | -0.04 | [-0.30, 0.21] | 0.02 | .733 | .866 | 0.02 | [-0.23, 0.27] | 0.02 | .866 | .866 |
| Rostral anterior cingulate | -0.06 | [-0.32, 0.19] | 0.02 | .624 | .832 | -0.02 | [-0.29, 0.23] | 0.02 | .829 | .866 |
| Posterior cingulate | 0.28 | [0.06, 0.58] | 0.01 | .036^a^ | .073 | 0.34 | [0.11, 0.58] | 0.01 | .005^a^ | .030^a^ |

*Note*. *β* = standardized coefficient, *CI* = confidence interval, *SE* = standard error of *β.* All *P* values were adjusted for multiple comparisons using the false discovery rate (FDR) method and a indicates *P* < .05.

**Table S4.** Associations between cyberbullying victimization at T1 and mental health outcomes/inhibitory control at T2 in the ABCD study after inverse probability weighting.

|  | *β* | 95% *CI* | *SE* | *P* | *P*_FDR_ |
| --- | --- | --- | --- | --- | --- |
| **Mental health outcomes** |  |  |  |  |  |
| Internalizing problems | 0.01 | [-0.18, 0.21] | 1.05 | .880 | .880 |
| Externalizing problems | 0.26 | [ 0.07, 0.45] | 0.86 | .007^a^ | .014^a^ |
| **SST behavioral performance** |  |  |  |  |  |
| Correct Stop Rate (%) | -0.02 | [-0.26, 0.21] | 0.01 | .830 | .830 |
| Stop Signal Reaction Time | -0.11 | [-0.30, 0.09] | 5.11 | .289 | .498 |

*Note*. *β* = standardized coefficient, *CI* = confidence interval, *SE* = standard error of *β.* All *P* values were adjusted for multiple comparisons using the false discovery rate (FDR) method and a indicates *P* < .05.

**Table S5.** Mediation effects of brain activation during error processing between cyberbullying victimization at T1 and externalizing problems at T2 in the ABCD study after inverse probability weighting.

|  | | Left hemisphere | | | | | | | | Right hemisphere | | | | | | |
| --- | --- | --- | --- | --- | --- | --- | --- | --- | --- | --- | --- | --- | --- | --- | --- | --- |
|  |  | Indirect effect | | 95% *CI* | | *P* | | *P*_FDR_ | | Indirect effect | | 95% *CI* | | *P* | | *P*_FDR_ |
| **Incorrect stop contrasted with correct go** | | | | | | | | | | | | | | | | |
| Superior parietal | 0.004 | | [-0.003, 0.006] | | .623 | | .934 | | 0.001 | | [-0.006, 0.005] | | .955 | | .955 | |
| Inferior parietal | 0.000 | | [-0.003, 0.004] | | .898 | | .955 | | -0.004 | | [-0.007, 0.002] | | .442 | | .934 | |
| Posterior cingulate | -0.005 | | [-0.007, 0.005] | | .579 | | .934 | | -0.006 | | [-0.008, 0.005] | | .601 | | .934 | |
| **Incorrect stop contrasted with correct stop** | | | | | | | | | | | | | | | | |
| Superior parietal | 0.003 | | [-0.002, 0.004] | | .709 | | .811 | | 0.003 | | [-0.001, 0.005] | | .626 | | .811 | |
| Inferior parietal | 0.003 | | [-0.002, 0.004] | | .811 | | .811 | | 0.006 | | [-0.001, 0.008] | | .758 | | .811 | |
| Posterior cingulate | 0.005 | | [-0.004, 0.006] | | .603 | | .811 | | 0.006 | | [-0.001, 0.008] | | .490 | | .811 | |

*Note*. *CI* = confidence interval. All *P* values were adjusted for multiple comparisons using the false discovery rate (FDR) method.

**Table S6.** Path coefficients in the longitudinal mediation analysis of cyberbullying victimization, brain activation during error processing, and externalizing problems in the ABCD Study after inverse probability weighting.

|  | Left hemisphere | | | | | | | | Right hemisphere | | | | | | | |
| --- | --- | --- | --- | --- | --- | --- | --- | --- | --- | --- | --- | --- | --- | --- | --- | --- |
|  | Path a | 95% *CI* | *P* | *P*_FDR_ | Path b | 95% *CI* | *P* | *P*_FDR_ | Path a | 95% *CI* | *P* | *P*_FDR_ | Path b | 95% *CI* | *P* | *P*_FDR_ |
|  |  |  |  |  |  |  |  |  |  |  |  |  |  |  |  |  |
| Superior parietal | 0.36 | [0.11, 0.61] | .004^a^ | .012^a^ | 0.01 | [-0.03, 0.06] | .574 | .861 | 0.34 | [0.10, 0.59] | .006^a^ | .012^a^ | 0.00 | [-0.04, 0.05] | .861 | .945 |
| Inferior parietal | 0.26 | [0.01, 0.51] | .041^a^ | .041^a^ | 0.00 | [-0.04, 0.04] | .945 | .945 | 0.33 | [0.08, 0.58] | .009^a^ | .014^a^ | -0.01 | [-0.06, 0.03] | .543 | .861 |
| Posterior cingulate | 0.28 | [0.03, 0.54] | .028^a^ | .034^a^ | -0.02 | [-0.06, 0.03] | .387 | .861 | 0.36 | [0.11, 0.61] | .005^a^ | .012^a^ | -0.02 | [-0.06, 0.02] | .365 | .861 |
| **Incorrect stop contrasted with correct stop** | | | | | | | | | | | | | | | | |
| Superior parietal | 0.32 | [0.10, 0.52] | .005^a^ | .015^a^ | 0.01 | [-0.03, 0.05] | .510 | .612 | 0.31 | [0.06, 0.48] | .010^a^ | .017^a^ | 0.01 | [-0.02, 0.05] | .397 | .612 |
| Inferior parietal | 0.30 | [0.08, 0.50] | .011^a^ | .017^a^ | 0.01 | [-0.03, 0.05] | .634 | .634 | 0.30 | [0.07, 0.51] | .014^a^ | .017^a^ | 0.02 | [-0.02, 0.06] | .486 | .612 |
| Posterior cingulate | 0.28 | [0.06, 0.58] | .036^a^ | .005^a^ | 0.02 | [-0.01, 0.06] | .199 | .612 | 0.34 | [0.11, 0.58] | .005^a^ | .015^a^ | 0.02 | [-0.01, 0.05] | .246 | .612 |

*Note*. *CI* = confidence interval. The path a is from T1 cyberbullying victimization to T2 brain measures and the path b is from T1 brain measures to T2 externalizing problems. All *P* values were adjusted for multiple comparisons using the false discovery rate (FDR) method and a indicates *P* < .05.

.
